# Artificial Intelligence Meets Micro/Nanorobotics

**DOI:** 10.1002/adma.74721

**Published:** 2026-08-27

**Authors:** Fatma M. Yurtsever, Minitha Cherukutty Ramakrishnan, Ian Kuula Ross, Ivan Zelinka, Hamed Shahsavan, Michal Otyepka, Martin Pumera

**Affiliations:** ^1^ Future Energy and Innovation Laboratory Central European Institute of Technology Brno University of Technology Brno Czech Republic; ^2^ Department of Chemical Engineering Institute For Polymer Research Center For Bioengineering and Biotechnology Waterloo Institute for Nanotechnology University of Waterloo Waterloo Ontario Canada; ^3^ Faculty of Electrical Engineering and Computer Science VSB – Technical University of Ostrava Ostrava Czech Republic; ^4^ IT4Innovations VSB – Technical University of Ostrava Ostrava Czech Republic; ^5^ Regional Centre of Advanced Technologies and Materials Czech Advanced Technology and Research Institute (CATRIN) Palacký University Olomouc Olomouc Czech Republic; ^6^ Office of Research and Development Asia University Taichung Taiwan; ^7^ Department of Chemical and Biomolecular Engineering Yonsei University Seoul South Korea

**Keywords:** biomedicine, closed‐loop autonomy, deep learning, digital twins, environmental remediation, machine intelligence, microrobots, nanorobots

## Abstract

Microrobots and nanorobots are a developing technology, which evolved from simple “motoric” motion‐capable micro/nanomachines to physical machine intelligence capable of communicating with each other using chemical or physical signals. Meanwhile, artificial intelligence (AI) is rapidly integrating into our modern life. We explore here how AI can be implemented in micro‐ and nanorobotic systems. The advancement of AI enhances micro‐ and nanorobots’ performance but also drives a fundamental transition from externally actuated, task‐specific platforms toward autonomous, adaptive, and multifunctional systems capable of operating in highly complex environments for clinical and environmental applications. At the micro/nanoscale, propulsion, sensing, and control face several limitations, including limited onboard computation, which hinder deterministic navigation and task execution. Recent advances in machine learning, including deep learning, reinforcement learning, and physics‐informed models, now offer powerful solutions to overcome those limitations. This perspective highlights how the convergence of AI and micro‐ and nanorobot technologies is creating a promising hybrid discipline for translational applications in both clinical and environmental settings. AI‐assisted design accelerates the discovery of functional materials tailored to specific applications, optimizes geometric and surface properties for efficient propulsion, and enables generative fabrication of micro‐ and nanorobot architectures. Moreover, deep learning‐based ultrasound, optical depth estimation, and multimodal perception enable precise real‐time localization—an essential requirement for closed‐loop autonomythereby enhancing the imaging and tracking of micro‐ and nanorobots. Additionally, the use of digital twins offers a predictive model of real‐world experimentation supporting risk assessment and patient‐specific planning for biomedical applications. The advances in AI‐enhanced micro‐ and nanorobots will transform the targeted drug delivery, minimally invasive diagnostics, biosensing, environmental remediation, and pollutant capture by integrating real‐time perception and adaptive decision‐making into fully autonomous devices in complex clinical and environmental conditions.

## Graphical abstract

Artificial intelligence is transforming micro‐ and nanorobots from externally controlled, task‐specific machines into adaptive, autonomous systems. Machine learning, multimodal perception, digital twins, AI‐guided materials and geometry design enhance propulsion, localization, decision‐making, whichaccelerates clinical and environmental applications including targeted delivery, diagnostics, biosensing, remediation, and pollutant capture.

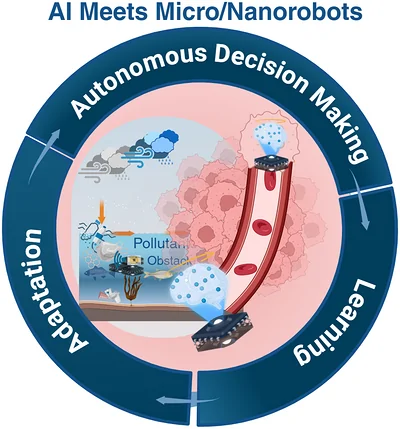

## 1 Introduction

Micro‐ and nanorobots are minuscule particles ranging from the size of a virus (10–20 nm) to several tens of micrometers and capable of motion and performing predetermined tasks or actions [1, 2, 3]. These nano and micromachines found the way to variety applications, ranging from biomedicine, [4] environmental remediation [5, 6] or food science [7]. Traditionally, they can be pre‐programmed chemically or physically, that is, by changing the physical or chemical environment around them; hence, such micro‐ and nanorobots show physical machine intelligence [8, 9, 10, 11]. Recently, machine intelligence/artificial systems were utilized to enhance the performance of these micro‐ and nanorobots [12]. Here, we summarize how recent advancements in AI can enhance micro‐ and nanorobotic technologies, including the limitations, advantages, and translations of real‐world applications.

Recent rapid advances in the field of artificial intelligence (AI) have become powerful enablers in engineering micro‐ and nanorobots for remote applications. AI‐assisted techniques facilitate complex tasks by leveraging data‐driven learning with machine learning (ML), deep learning (DL), and reinforcement learning (RL) for decision‐making, real‐time perception, and adaptive control strategies [13, 14]. The implementation of AI‐assisted micro/nanoscale robots in biomedical and remote environments poses significant scientific and engineering challenges. At the macroscale, incorporation of sensors and physics‐based models can help estimate the system state with high accuracy in the presence of noise. More importantly, it enables seamless integration with computers, software, and sensors; however, at the micro/nanoscale, this is far more challenging due to limited sensing bandwidth, optical/acoustic contrast, thermal fluctuations, and stochastic perturbation (*e.g*., Brownian motion), which substantially increase the state estimation uncertainty and complicate the feedback control [1, 3, 15, 16]. Thermal fluctuations and Brownian forces introduce stochasticity that must be modeled probabilistically while limited sensing resolution and sampling rates further constrain state estimation. Consequently, control and navigation are naturally formulated in terms of probabilistic state inference and decision‐making under uncertainty, where learning‐based models can complement physics‐based dynamics. This is a “middle region,” where certainty of both momentum and position, or simply trajectory, is not possible. AI adoption at small scales reflects a shift in methodology: from purely model‐based, deterministic control pipelines toward hybrid approaches that combine first‐principles dynamics with probabilistic state estimation and data‐driven policies under partial observability (*e.g*., stochastic control, Bayesian filtering, and reinforcement learning). External forces and reactions taking place in micro‐ and nanorobotic fluidic media, such as blood vessels or body lumens, define chaotic and noisy environments that are often unmeasurable. The combination of onboard (embodied) and off‐board intelligence that drives micro‐ and nanorobots enables them to solve problems inductively, akin to neural turing machines (NTMs), mostly by recognizing patterns [1, 17]. This hybrid architecture allows the system to manage the uncertain accuracy inherent in stochastic fluidic and confined micro‐environments by combining rational off‐board control algorithms with embodied intuitive, pattern‐based responses. In this context, the behavior of micro‐ and nanorobots enhanced by AI will no longer be strictly bound to predefined conditions as they can gain information directly from their biological or environmental media.

In this framework, AI has emerged as a game‐changing technology, becoming a necessity for revolutionizing technologies that are intertwined with human life. By leveraging numerous algorithms of ML, DL, and RL, AI has reshaped the autonomous control of nanorobots by allowing them to compensate for physical constraints, adapt to complex environments, extract information for precise tracking, and perform multiple tasks [18, 19, 20]. Hence, AI‐enhanced micro‐ and nanorobotics is projected to converge, marking a departure from externally actuated systems and opening new roads for autonomy at small scales.

Micro‐ and nanorobots typically depend on propulsion strategies such as an external magnetic field, chemical‐driven, light‐driven, and ultrasound actuation mechanisms [21, 22, 23, 24, 25, 26, 27]. However, accurate locomotion at these scales is inherently challenging. Physics of motion, like what is relevant in traditional robotics, can no longer be applied to micro‐ and nanorobots due to the low Reynolds number of surrounding fluids and trajectory instability caused by Brownian motion [28].

In any actuation mechanisms, variation in the control field over space is determined by the spatial structure of the applied field. A spatially uniform field acts globally, applying a near‐uniform control signal over the entire workspace simultaneously. Consequently, localized control fields for steering, focusing, or individually addressing specific regions of the workspace require non‐uniform fields with non‐zero gradients or higher‐order spatial derivatives. This structure is described compactly by expressing the control field as a multipole expansion of its scalar potential. Starting with a uniform scalar monopole term, followed by a uniform vector dipole term, with quadrupole, hexapole, and higher order multipole terms describing localization and spatial variation within the control field. As a robot shrinks toward the nanoscale, the spatial span of the control signal across its body diminishes. The field becomes effectively uniform over the body, and the interaction collapses onto its leading directional term. Localization, therefore, cannot be resolved across an individual nanorobot; it is imposed by the multipole structure of the field distributed across the workspace. Magnetic control systems like the OctoMag already exploit this separation directly. Although magnetic fields have zero monopole moment, their dipole moment describes the uniform alignment imposed by magnetic torque ***τ*** = **m** × **B** across the entire workspace. Quadrupole, hexapole, and higher‐order multipole moments introduce the necessary spatial variation required for translational force ***F*** = ∇(**m**·**B**) via magnetic field gradients. By combining multipole terms up to a given order, multiple‐degree‐of‐freedom control can be achieved [29, 30] with each additional degree of freedom increasing the achievable complexity of localized patterning that can be achieved across the workspace.

Energy and power are two of the fundamental limitations for AI‐assisted micro‐ and nanorobotic systems due to the lack of power storage and limited computation in micro‐ and nanoscale. Even though the use of potential fields can easily overcome the issue of limited on‐device computation, power still remains a limiting constraint, although in a somewhat different form. Generating strong, highly localized fields imposes practical power and thermal budgets on the actuation hardware, which constrain achievable localization in confined biomedical settings.

Finally, factors such as environmental viscosity and tissue heterogeneity limit the efficiency of micro‐ and nanorobots in attaining deterministic repeatability in open‐loop control methods [31]. Hence, there is a critical need for closed‐loop control with continuous feedback. Figure 1a presents the challenges where AI offers a powerful solution when data‐driven models are used to visualize system dynamics, familiarize themselves with propulsion strategies, and optimize control policies, even under uncertain or noisy conditions [32, 33, 34, 35]. AI‐assisted systems utilize learning algorithms and optimization during the design phase, while utilizing learning algorithms during the actuation and navigation phases control and adapt the policies. Therefore, AI does not only act as a controller of micro‐ and nanorobots but also to design the material, morphology, and size of the micro‐ and nanorobots [23, 36].

**FIGURE 1:**
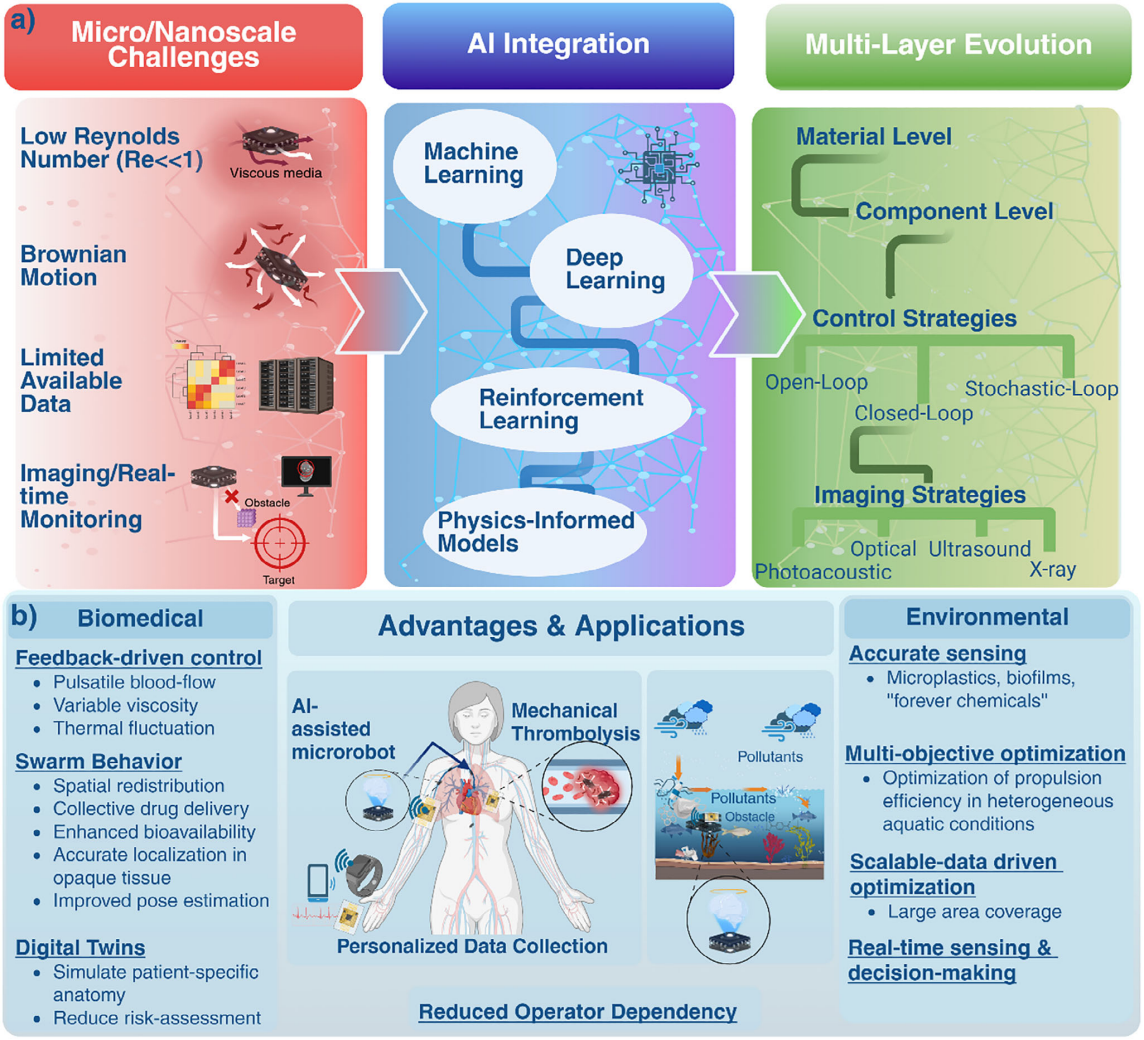
Conceptual framework of AI‐assisted micro‐ and nanorobotics. a) Micro‐ and nanorobots operate under severe limitations, including low Reynolds number hydrodynamics due to a viscous microenvironment (Re ≪ 1), Brownian motion, limited available dataset, and scarcity of real‐time imaging and monitoring for small‐scale micro‐ and nanorobots. Integrating artificial intelligence (AI) into micro‐ and nanorobots promises data‐driven solutions to overcome these limitations by enabling adaptive control and decision‐making based on machine learning, deep learning, reinforcement learning, and physics‐informed models. On the other hand, simultaneous multi‐layer evolution in AI‐assisted material discovery and component‐level optimization, advancing control strategies, and integrating the multi‐modal imaging help accelerate the advancement in AI‐assisted micro‐ and nanorobotics. b) AI‐assisted micro‐ and nanorobots enable autonomous and precise operation in biomedical applications such as mechanical thrombolysis and environmental applications, including pollutant and microplastic detection, capture, and removal while reducing operator dependency and enabling scalable, data‐driven optimization. https://BioRender.com/d7xnpt0.

The next generation of micro‐ and nanorobots would be ideally capable of intelligently interacting with their surroundings and making decisions relevant to the environment, thereby improving adaptability in biomedical conditions such as complex vasculature, organs, pathological sites, and intelligently detecting pollutants in the complex environmental realm as shown in Figure 1b [19]. Micro‐ and nanorobots cannot rely exclusively on physical robustness like a macroscale robot; instead, intelligence must be rooted in algorithms that continuously learn and adapt. AI‐driven micro‐ and nanorobots have already shown autonomous swarm distribution [37] and multimodal imaging‐based localization [32, 38].

AI enhances autonomy by enabling perception–decision–actuation loops under uncertainty and improving tracking, planning, and control while explicitly quantifying confidence and failure modes rather than assuming error‐free analysis. Magnetic micro‐ and nanorobots combined with reinforcement learning improve 3D positional control [18]. Also, explainable deep learning models facilitate the classification and localization of micro‐ and nanorobots using ultrasound imaging [39]. Machine learning frameworks now support the fabrication of 3D‐printed micro‐ and nanorobots for biomedical applications while digital twins simulate microfluidic interactions for real‐time optimization [40, 41]. Unlike conventional micro‐ and nanorobots, which are often task‐limited and environment‐agnostic, AI‐driven micro‐ and nanorobots should be able to integrate perception, decision‐making, and actuation into cohesive, adaptive frameworks. These properties not only improve control but also serve as a paradigm shift in how intelligent systems are conceptualized at such small scales.

## 2 AI for Design and Material Optimization for Micro‐ and Nanorobots

The inherent limitation of micro‐ and nanorobots for the installation of complex processing units necessitates the inclusion of an embodied intelligence, which tightly couples the micro‐ and nanorobot body to an external control system (off‐board intelligence) and its environment. Via embodied intelligence, a large number of the calculations necessary for the function of micro‐ and nanorobots are offloaded, for example, morphological computation, to the body of the micro‐ and nanorobot itself. For instance, micro/nanorobots fabricated from compliant polymeric materials can take advantage of their material properties and their interactions with the surrounding medium to produce adaptive responses. At these length scales, such behavior is typically governed by the robot's geometry, mechanical characteristics, and interactions with the local environment, rather than by the large, actively controlled deformations commonly observed in macroscale soft robots. In this view, the design of micro‐ and nanorobots requires optimization of a large number of parameters, ranging from building block materials chemistry, geometry, surface properties, biocompatibility with surrounding fluids, propulsion mechanism, function, and efficiency. Such optimization is a grand and longstanding challenge of micro‐ and nanorobots, dramatically limiting their large‐scale and cost‐effective production for desired applications. AI‐driven data‐guided computational strategies are now reshaping the way micro‐ and nanorobots are designed and fabricated.

A theoretical multi‐level evolution (MLE) framework for the development of next‐generation robots with embodied intelligence has been reported. This framework is inspired by the evolution of biological organisms, where molecular‐level units, like DNAs and proteins, and events involving them, cascade upward to define macro‐level limbs or body forms via morphogenesis [42]. MLE is based on a bottom‐up design process across three hierarchical levels that fuel robot designs: material‐level, component‐level, and robot‐level. MLE approach enriched with AI can unlock the complex nature of the design and fabrication of micro‐ and nanorobots. It operates within a “design loop,” which iteratively validates robotic designs from predicting the candidate materials and forms to ensuring integrated robots are viable for the intended application. In contrast to traditional optimization that seeks the best solution, AI‐driven MLE in micro‐ and nanorobotic systems could particularly benefit from quality–diversity (QD) optimization algorithms that offer a wide library of solutions. This approach ensures that if an optimal design fails, another functional alternative can be chosen from the library [43].

At the material‐level, the discovery and optimization of micro‐ and nanorobots’ molecular and material building blocks can be tackled by AI algorithms, such as those already used in materials informatics [44] and discovery, [45, 46] to identify candidates with specific properties, such as biocompatibility, magnetic responsiveness, or stimuli‐responsive shape morphing. Data‐driven frameworks enable the prediction of biocompatible coatings, anti‐fouling surfaces, and environment‐selective surface chemistry. For instance, recent work has shown that how imine‐functionalized mesoporous magnetic silica nanoparticles optimized with ML demonstrate enhanced pollutant removal efficiency [23, 24]. At the component‐level, building block materials are formed into certain geometries to create functional components like sensors, actuators, and structural elements. AI, for instance, can guide the component‐level design to define a helical geometry for magnetoactive materials to enable them with flagellar‐inspired corkscrew propulsion, a design superior to simple spherical aggregates in viscous fluids [47]. Machine learning algorithms can categorize optimum morphologies for propulsion under low Reynolds number conditions, balancing thrust efficiency and environmental adaptability [33]. In parallel, generative design algorithms simplify the creation of innovative designs for 3D‐printed micro‐ and nanorobots with tailored functionalities, such as cargo attachment sites or chemical sensing modules [40].

At the robot‐level, components are integrated into a robotic form that allows for performance on a specific task within a simulated or physical environment. Here, AI can optimize the assembly of components to ensure coherent system behavior, utilizing evolutionary algorithms to refine robot forms over successive generations. These findings suggest that AI serves not only as a control mechanism but also as a design accelerator, shaping the physical characteristics of next‐generation micro‐ and nanorobots [36].

## 3 AI for Imaging, Localization, and Tracking

Current approaches to the targeted tracking of micro‐ and nanorobots in complex biological or environmental settings remain a significant challenge, and much of the research aimed at improving them still relies on iterative experimentation. Investigation using optical microscopy‐based navigation is constrained by shallow penetration depth, nanorobot tracking resolution, and sensitivity to illumination intensity. Magnetic resonance and ultrasound can image deeper regions but often lack the spatial precision required to localize individual micro/nanorobots. A further challenge arises when imaging and actuation must be performed simultaneously. Imaging systems require strong interactions between the applied field and both the robot and its surroundings to generate sufficient contrast, whereas the actuation is most effective when the robot responds selectively to the applied field while the surrounding medium remains largely unaffected. Both require high sensitivity and high selectivity (sensitivity being how much of an imaging field's energy a micro/nanorobot can respond to, and selectivity being the ratio of micro‐ and nanorobots’ sensitivity to the environment's sensitivity). High‐frequency driving/imaging fields (radio frequency‐RF, optical, ultrasound) typically have high sensitivity, but low selectivity. This makes them well‐suited for sensing (as they image both micro‐ and nanorobots and their surroundings) but poorly suited to manipulation (as the reaction of the surroundings to the field is also significant). In the same vein, a low selectivity toward the driving/imaging field can also make it difficult to resolve micro‐ and nanorobots with their surroundings. Therefore, it can use fields with low sensing selectivity to image the entire system but use fields with high selectivity to manipulate or acquire positional feedback. These can be entirely different fields (*i.e*., ultrasound for imaging and magnetic for control) or the same field at different frequencies (*i.e*., optical imaging and magnetic control). Recent advances in AI‐driven imaging strategies have shown the potential to overcome these hurdles and limitations [48, 49, 50].

Deep learning algorithms now enable real‐time pose and depth estimation of optical micro‐ and nanorobots, even in highly scattering environments where conventional imaging processing methods lose tracking accuracy. Lan Wei et al. [51] reported a set of deep learning models for training and evaluating microrobot pose and depth estimation using an optical microrobot dataset. The dataset was generated using an optical tweezers platform integrated with a bright‐field optical microscope and comprises 232,881 number of images collected from 18 optically manipulated microrobot designs across 176 pose configurations. The authors compared eight deep learning architectures, and vision transformer‐based models achieved the highest accuracy for pose estimation. These studies on optical microrobots focus on localization and tracking, with quantitative benchmarks for evaluating learning‐based perception methods. J. Lin et al. [52] reviewed recent developments in magnetically actuated microrobots designed for in vivo cargo delivery. Depending on the target tissue and experimental conditions, robot motion was monitored using ultrasound, magnetic resonance imaging, x‐ray fluoroscopy, optical microscopy, or photoacoustic imaging. Integrating magnetic actuation with complementary imaging techniques enables continuous localization of the microrobot and supports closed‐loop navigation during cargo delivery. Physics‐informed neural networks integrate physical models with adaptive ML frameworks to improve orientation and position estimation [53]. The experiments were conducted using an optical tweezers system integrated with a bright‐field optical microscope, where microrobots were translated along the optical axis to acquire image sequences at different focal depths. This reduced depth estimation errors, particularly near focal‐plane transitions, and improved the robustness of axial localization, providing more reliable feedback for closed‐loop optical microrobot manipulation. Furthermore, explainable deep learning models have been developed for automated ultrasound‐based classification and localization of micro‐ and nanorobots, providing robust feedback during in vivo studies, which allows transparency and helps explain model decisions with visual explanations [39]. These advances enhance high‐precision monitoring and enable closed‐loop control by directly integrating data into navigation algorithms. Such capabilities are vital for clinical translation, where micro‐ and nanorobots must be accurately tracked within opaque tissues and organs.

AI‐enhanced imaging frameworks can integrate with propulsion control to support simultaneous navigation and sensing, allowing micro‐ and nanorobots to act as both carriers and sensors. This integration ensures accurate, real‐time responses to environmental changes, a feature that is crucial for both biomedical applications (*e.g*., targeted drug delivery) and environmental applications (*e.g*., pollutant detection and collection).

## 4 AI for Navigation, Control, and Swarm Intelligence

The navigation of micro‐ and nanorobots through dynamic and heterogeneous environments is possibly the most exciting and challenging aspect of micro‐ and nanorobots’ operation. Current navigation control methods rely on open‐loop commands, which are inherently limited in environments with dynamic microenvironments and thermal fluctuations that result in unmeasured disturbances (*e.g*., flow variability) and dominate the motion. In open‐loop (Figure 2a) or predictive control, the state of the system is predicted using a feed‐forward function (*e.g*., actuation) with the assumption that external reactions are negligible, making them well‐suited to systems in stable equilibrium. In closed‐loop (feedback) control (Figure 2b), measurements are inherently noisy and often indirect; therefore, state observers (*e.g*., Kalman/particle filters) and robust control designs are used to estimate the relevant variables and stabilize performance around a set‐point. For micro‐ and nanorobots, noise measurement, limited imaging contrast, latency, and Brownian perturbations can dominate, making probabilistic estimation and stochastic/robust control formulations particularly appropriate. The “error” term (the difference between set point and measurement point) is never at a single, determinable value. It can only control micro‐ and nanorobots exactly within a minimum range of measurement uncertainty. Below this point, a deterministic control scheme cannot be used, and statistical ones become advantageous. This approach to modeling navigation is well‐suited to current approaches to AI. A practical strategy is to start with a physics‐based dynamic model of actuation and hydrodynamics and then learn data‐driven residuals that capture unmodeled effects (*e.g*., local flow disturbances, imaging‐induced biases, or fabrication variability). A suitable abstraction is a partially observable stochastic control problem (POMDP), where the controller maintains a belief over the robot state and learns uncertainty‐aware policies (*e.g*., via RL/DRL) in simulation and transfers them to real systems with online calibration. This hybrid modeling approach reduces sample complexity and improves generalization compared with purely black‐box learning. Therefore, the general approach would be to start with a reasonable “ab initio” model that outlines the intended actuation mode and has a stochastic “diffusion term,” which is initially assumed to be Brownian diffusion. In stochastic control (Figure 2c), at least one important variable cannot be known with certainty. This variable can be the position, velocity, and so forth, of the micro‐ and nanorobots. The state is modeled as a weighted distribution of possible values. Each micro‐ and nanorobot can be represented by a belief distribution (a probabilistic state estimate) over its possible states. Live measurements are fed to a training model; if the measured values are certainly off, then they provide negative feedback. Once errors become ambiguous, the control module regresses measurements to find patterns to update itself from learned patterns. With this, the model is well‐suited for adaptive training. The error does not converge to zero as in closed‐loop control but becomes an error distribution. Biases in this distribution can be used to “calibrate” control modalities (i.e., remove a wobble from a helical swimmer). Artificial intelligence, particularly reinforcement learning (RL) and deep reinforcement learning (DRL), has enabled micro‐ and nanorobots to adopt adaptive navigation strategies that surpass these limitations [54]. Reinforcement learning (RL) frameworks have demonstrated notable success in achieving 3D positional control of magnetic micro‐ and nanorobots [18]. In these systems, the robot acquires navigation strategies over trial‐and‐error interaction with simulated or real environments, allowing it to adapt to unpredictable flow conditions. Similarly, deep reinforcement learning has been applied to magnetically driven micro‐ and nanorobots, enabling them to “learn to swim” under varying fluid dynamics [55].

**FIGURE 2:**
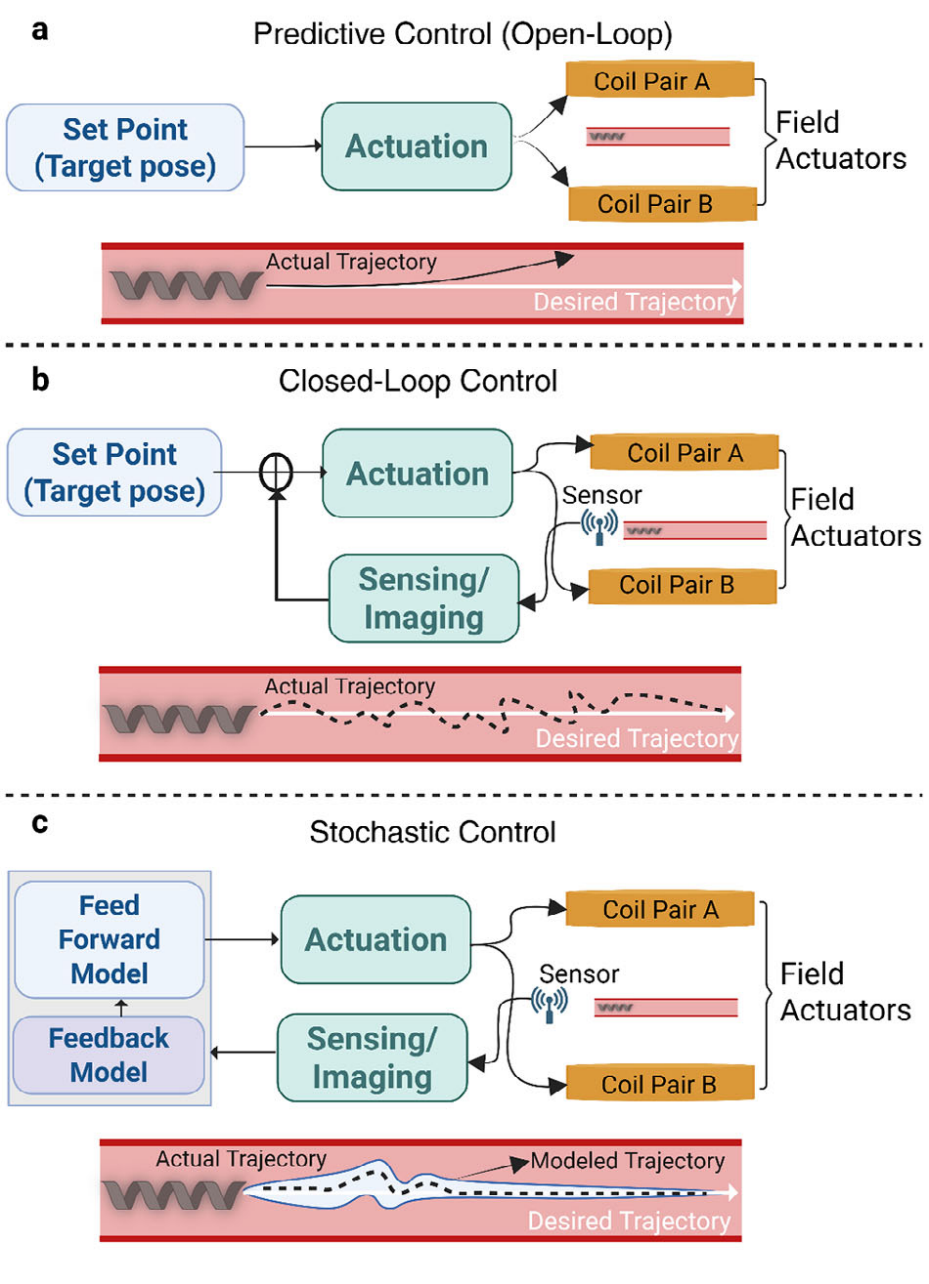
Conceptual schematic representation of a) an open‐loop control system, b) a closed‐loop control system, and c) a stochastic control system. In practice, closed‐loop and digital twin frameworks typically include online state estimation, model adaptation, and predictive optimization layers. https://BioRender.com/nmimj2i.

Swarm control and swarm intelligence describe phenomena that may appear similar at the macroscopic scale but arise through completely different mechanisms. Swarm control assumes that micro/nanorobots coupling to the external field dominates, causing top‐down organization of the swarm's individual members. Under swarm control, the entire swarm system may be treated as numerous copies of a single micro/nanorobot, highlighting the compatibility between swarm and stochastic control techniques. Stochastic control can be thought of as controlling a swarm of possible states. Swarm intelligence, conversely, assumes that inter‐robot coupling dominates. The external field still organizes micro/nanorobots, but this is secondary to organization between them and their immediate neighbors [56]. This strong coupling between neighbors allows the emergence of bottom‐up organization of micro/nanorobot swarms into unique “phases,” with each phase often possessing unique kinematic or morphological properties. For magnetic microrobot systems, the spontaneous organization of liquid, chain, vortex, and ribbon states can be observed after applying different alternating magnetic fields. By cataloguing the organizing field associated with each emergent phase, the swarm morphology, behavior, and function can be modified at runtime to suit different deployment scenarios [57]. The contrasting case is intelligence compiled into a single body before deployment: in liquid crystalline elastomers, molecular anisotropy is patterned and then locked in by polymerization, so that shape change and locomotion follow from the material's own programmed structure rather than from a runtime controller [58, 59].

Beyond single‐robot navigation, AI has facilitated autonomous swarm coordination. Deep learning models support real‐time distribution planning, allowing micro‐ and nanorobot collectives to dynamically divide, combine, or redirect in response to environmental cues [37]. These methods reduce dependence on external operators and enable scalable arrangements for large‐area tasks such as tissue penetration or remote pollutant removal.

Human‐in‐the‐loop approaches further enhance learning, where micro‐ and nanorobots acquire complex navigation skills from human demonstrations via imitation learning [60]. This strategy accelerates the transfer of advanced decision‐making to autonomous micro‐ and nanorobots, blending human intuition with machine adaptability. Figure 3 represents examples of next‐generation AI‐incorporated micro‐ and nanorobots, including magnetic actuation in Figure 3a(i),b(i) with learning algorithms, including reinforcement learning (Figure 3a) and deep learning (Figure 3b). The respective trajectories are shown in Figure 3a(ii),b(ii).

**FIGURE 3:**
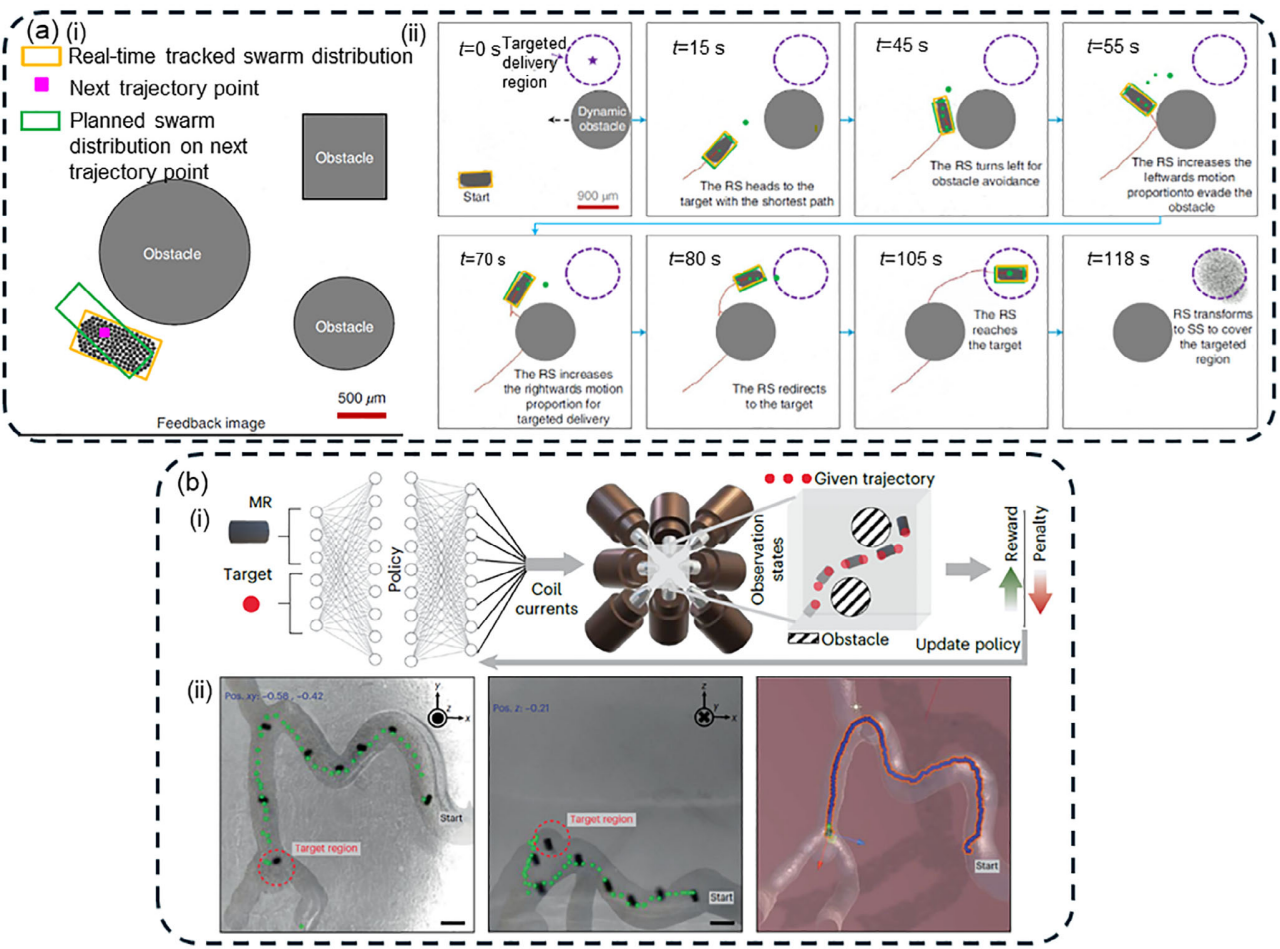
AI‐assisted actuation and control of micro‐ and nanorobots in complex environments. Representative examples from literature show (a) deep neural network (DNN)‐implemented micro‐ and nanorobots for real‐time decision‐making for swarm shape planning (SSP) and swarm orientation planning (SOP). (i) Real‐time swarm tracking and trajectory planning in an arena containing multiple obstacles. Color overlays indicate measured swarm distribution, planned distribution, and the next trajectory point. (ii) Time‐lapse sequence demonstrating obstacle‐aware navigation toward a target using DL‐informed control, including directional corrections and swarm redistribution. Scale bars: 500 µm. Reproduced with permission [37]. Copyright 2022, Springer Nature. (b) Reinforcement learning (RL)‐based magnetic field optimization for targeted motion in complex environments. (i) Magnetic field generator setup generates currents to drive micro‐ and nanorobots’ motion while avoiding obstacles, with rewards assigned according to trajectory adherence. (ii) 3D visualization using a brain phantom model with an aneurysm. Experimental demonstrations of RL‐assisted micro‐ and nanorobot navigation through tortuous paths in vitro, including obstacle avoidance and accurate arrival at a predefined target region. Trajectories (colored lines) illustrate real‐time instant corrections. Scale bars: 2 mm. Reproduced with permission [18]. Copyright 2024, Springer Nature.

Traditional micro‐ and nanorobots usually depend on open‐loop control, where pre‐programmed commands direct motion without feedback. Such strategies are limited in volatile environments, where instabilities rapidly degrade performance. AI enables the transition to closed‐loop, feedback‐driven autonomy where micro‐ and nanorobots continuously vary their motion based on sensory input and predictive models [31, 61]. For example, model‐based reinforcement learning has been introduced to ultrasound‐driven micro‐ and nanorobots, integrating real‐time feedback for adaptive track planning [23]. Similarly, AI‐enhanced nonlinear control frameworks have been applied to microrobot navigation in pulsatile blood flow, where adaptive algorithms evaluate dynamic disturbances in real time [62]. This evolution from open‐loop to adaptive, closed‐loop autonomy is a fundamental step toward fully autonomous microrobotics. It enables micro‐ and nanorobots not only to tolerate but to flourish in highly variable, noisy, and dynamic biological environments.

In a real‐time scalable solution, some functionalities of a single micro‐ and nanorobot become limited by external physical constraints and efficiency. These inherent limits micro‐ and nanorobots’ performance in biomedical applications. The concept of swarm intelligence provides a scalable solution, where large groups of micro‐ and nanorobots coordinate to achieve collective outcomes that surpass individual capabilities. Inspired by biological living systems such as bacterial colonies and ant collectives, AI‐assisted swarm control has become a significant technique in micro‐ and nanorobots. Environment‐adaptive swarms guided by AI algorithms can autonomously change their spatial arrangement to avoid obstacles and increase delivery efficiency [37, 63]. AI‐assisted swarm control is a vital feature for biomedical applications such as targeted therapy. Swarms of nanorobots can penetrate deep tissue layers more effectively than a single device, enabling collective tasks, enhancing local drug delivery at the cellular level, and distributing across large target sites [31, 63]. Understanding and controlling swarm behavior is highly crucial for environmental applications. Swarm behavior allows a large population of micro‐ and nanorobots to work collaboratively to maximize coverage of the targeted field. AI‐enhanced micro‐ and nanorobots may also be capable of real‐time sensing and generating predictive pollutant dispersion models.

Actuation under real physiological conditions, including blood flow, viscous forces, and thermal fluctuations, may restrict the precisely controlled motion of micro‐ and nanorobots. It has been reported that AI‐enhanced actuation of magnetic micro‐ and nanorobots, including an adaptive backstepping controller with weight estimation, provides estimation of the behavior in pulsatile blood‐flow conditions [62]. Currently, the actuation of magnetic micro‐ and nanorobots is based on the assumption of a homogeneous magnetic field, environment, and the micro‐ and nanorobots’ properties, without consideration of their adaptability. It has been demonstrated that the reinforcement learning‐based actuation of micro‐ and nanorobots minimizes delays of their independent control [18]. Reinforcement learning can be used to fine‐tune propulsion or actuation parameters in response to fluctuating flow conditions.

Optical micro‐ and nanorobots, which require light to actuate and guide micro‐ and nanorobots, have emerged as a powerful strategy for precise, non‐invasive control. Reliable depth estimation is crucial for closed‐loop‐control control with the ultimate goal of avoiding damaging surrounding biological tissues. Feature‐based deep learning has been used as a traditional method for pose and depth estimation. However, there are several challenges, including inaccurate depth estimation in scattering media such as biological tissues caused by rapid environmental changes during manipulation, low contrast imaging under a microscope, a lack of available large‐scale datasets, and environmental noise, which limit the extraction of distinct depth estimation. When large, labeled datasets are unavailable, depth/pose estimation can still be learned by leveraging physics‐based image‐formation constraints and weak or self‐supervised signals. In practice, focus/quality proxies (*e.g*., Laplacian‐of‐Gaussian responses, image–entropy measures, or gradient‐based sharpness metrics) can be used as auxiliary objectives or regularizers rather than being treated as datasets [51, 53].

In parallel, AI‐enhanced imaging frameworks integrated with propulsion control can leverage accurate and precise therapeutic delivery to adapt their locomotion in response to visual feedback [32, 33]. Deep learning‐enhanced imaging enables simultaneous navigation and diagnosis, where micro‐ and nanorobots act as both carriers and sensors within biological systems.

## 5 Digital Twins and Predictive Modeling

One of the most transformative paths of AI‐enhanced micro‐ and nanorobots is the development of digital twins, where the model is a “virtual” copy of the physical system (Figure 4). Representation can be abstracted and/or simplified. The physical system receives a command or set‐point and then controls the function. The information of the physical system can be measured through sensors. The physical system is then linked to its virtual copy. External powers provide physical stimulus to micro‐ and nanorobots. Sensors measure effects of stimulation and surrounding objects, where micro‐ and nanorobots simulate their interactions with complex physiological conditions in real time. Digital twins enable *predictive simulation and decision support* by maintaining a synchronized virtual representation of the physical system. By combining real‐time sensing with model updating, the twin can forecast trajectories and uncertainties, evaluate candidate actuation sequences, and support risk assessment and patient‐/site‐specific planning before and during operation.

**FIGURE 4:**
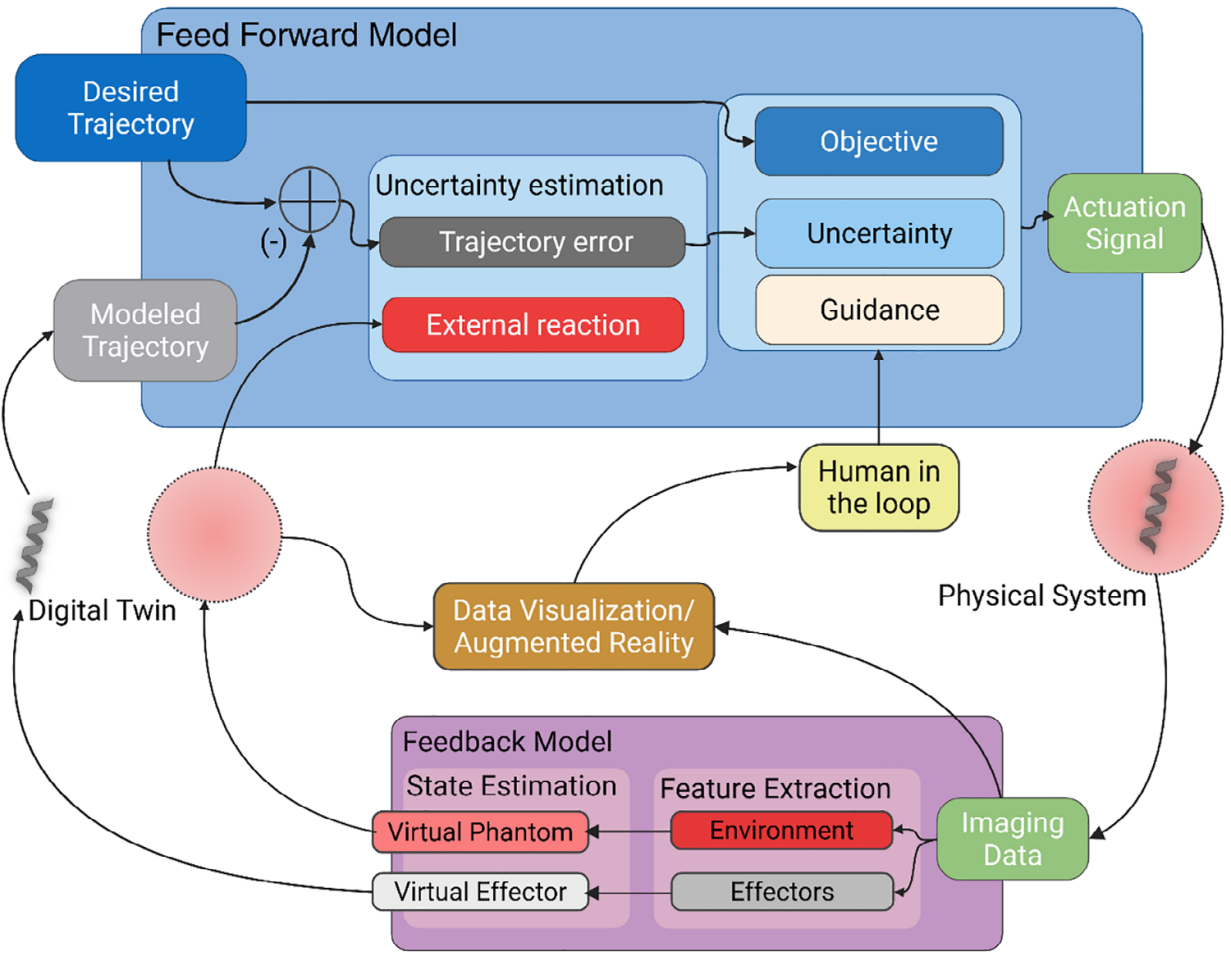
Conceptual schematic representation of a digital twin. The feedback process (violet box, bottom) serves to parse imaging data from the physical system, leveraging segmentation, classification, and other computer vision algorithms to estimate the state of the effector and its surroundings updating the digital twin. The feed‐forward process (blue box, top) uses the evolution of the digital twin model to estimate error and uncertainty from the target trajectory to incorporate into the planned trajectory. The human in the loop serves to assess the state of both the physical system and twin, providing guidance for decisions that either cannot or should not be trained, such as risk assessment. https://BioRender.com/1dyn60e.

It was demonstrated that digital twins can serve as a preoperational stage for micro‐ and nanorobots and thereby allow the simulation of real patient coordinates under x‐ray fluoroscopy [34]. By integrating the digital twin systems with real‐time medical imaging, clinicians can visualize micro‐ and nanorobot actuation in vivo and predict potential outcomes, thus enabling preemptive adjustments to trajectories or interventions [64]. Future of implementation of digital twins into the biomedical application of the microrobots is expected to incorporate multi‐modal imaging combining magnetic resonance imaging, ultrasound imaging, computer‐aided tomography, and/or x‐ray fluoroscopy. Incorporation of multi‐modal imaging into digital twins will enable continuous data collection from the virtual modal, while real‐time sensing and monitoring of the localization and state estimation [32, 65]. Further developments in image‐based 3D reconstruction and adaptive grid‐based learning strengthen the application of digital twins for micro‐ and nanorobots [41, 51]. This approach not only reduces experimental costs and animal testing but also accelerates clinical translation.

At the micro/nano scale, physical interactions that drive real systems are not completely known, especially when such systems have biological components. Regardless of the architecture, all model training fundamentally boils down to statistical regression or using linear algebra to identify patterns within training data. These patterns provide heuristic shortcuts that allow a model to quickly infer or predict a system that may be otherwise too complex, or even impossible to solve computationally. This poses a unique challenge for the use of AI in biomedical micro/nanorobotics: the microscopic interactions that drive our system are simultaneously complex and difficult to measure. Large, robust datasets for microscopic robots’ interactions and biological processes are not available for regression. Because of this, it is advantageous to treat each digital twin as the combination of two separate models, one is dedicated to describing reactions between the robots and the control field, and another separate model to describe the robots’ physical environment. Decoupling these models allows for each of these complex systems to be approached independently. By splitting the digital twin into two separate “domains,” the relevant dataset for training each sub‐model is greatly expanded. Instead of training on the available data for a target micro/nanorobot and organ system simultaneously, each sub‐model can be trained on any available data for the desired robot system in any environment, or the desired organ system with or without the presence of any effector system. With this, we temporarily forego the aim of training a digital twin for our specific system in pursuit of a “world model:” a generalized model which effectively distributes the discovery of complex interactions like fluid mechanics, protein binding, or micro/nanorobot coupling over many different similar systems [66, 67]. In this way, training a generalized world model is the digital twin analogue to the process of pretraining in large language models. Both cases allow the cost of identifying very complex, but general phenomena to be shared across a wide range of possible applications, yielding a “checkpoint” or “sandbox” through which specific deployments of effector and organ systems can be fine‐tuned to cheaply produce digital twins from relatively small datasets for any one target deployment. Model‐based reinforcement learning has already been applied in exactly this form to ultrasound‐driven microrobots, achieving high navigation success from limited training and generalizing across vasculatures [23].

The integration of swarm intelligence, optical depth estimation, propulsion optimization, and digital twins makes a remarkable impact on the evolution of micro‐ and nanorobotics toward proactive, adaptive intelligent systems. Digital twins function with continuous state estimation rather than static simulations, which allows them to perform with minimal disturbance and uncertainty [31]. AI enables micro‐ and nanorobots to learn from prior experiences, generalize across diverse environments, and coordinate in real time with other micro‐ and nanorobots or digital replicas with DL and RL [33]. These learning models enable bidirectional communication from the physical object layer to the digital object and allow real‐time decision making [68]. The advancing the integration of the learning systems into the digital twins will be transformative for modern cyber‐physical systems which are summarized in Table 1 [69]. However, challenges remain, including real‐time synchronization between physical and virtual micro‐ and nanorobots, computational constraints, and the need for high‐resolution imaging data. Overcoming these limitations is essential to fully leverage digital twins for autonomous micro‐ and nanorobot operation. Other limitations will be addressed in the following sections.

**TABLE 1:** Recent advances in implementation of AI in micro/nanorobots according to functions, methodology, application, and contribution in micro/nanorobots.

AI functions	Representative study	AI methodology	Application	Contribution of AI in Micro/ Nanorobots	Refs.
Material discovery	Materials informatics for functional robotic materials	Machine Learning	Material Design and selection	Accelerated discovery of magnetic and biocompatible materials	[44, 45, 46]
Material and design optimization	AI‐guided optimization of imine‐functionalized mesoporous magnetic silica nanorobots	Machine learning, multi‐objective optimization	Environmental remediation	Optimized material composition and pollutant removal efficiency	[23, 24]
Imaging and state estimation	Optical microrobot pose and depth estimation	Deep learning	Closed‐loop optical manipulation	Real‐time pose and depth estimation from microscopy images	[51, 76]
Imaging and localization	Explainable ultrasound localization of microrobots	Explainable deep learning	In vivo navigation	Automated localization and interpretation of ultrasound images	[39]
Physics‐informed perception	Physics‐informed neural networks for localization	Physics‐informed neural networks	Closed‐loop control	Improved position and orientation estimation under noisy conditions	[53]
Navigation and control	Reinforcement learning for magnetic microrobot navigation	Reinforcement learning	Targeted navigation	Autonomous obstacle avoidance and 3D navigation	[18]
Adaptive control	Model‐based reinforcement learning for ultrasound microrobots	Model‐based RL	Biomedical navigation	Navigation with limited training data and improved generalization	[23]
Swarm intelligence	Deep‐learning‐based swarm distribution planning	Deep neural networks	Collective transport and delivery	Real‐time swarm formation and obstacle avoidance	[37]
Digital twins	Learning from human demonstrations	AI‐assisted predictive modeling	Endovascular navigation	Patient‐specific preoperative planning	[34]
	Adaptive grid learning and image‐based reconstruction	Machine learning	Digital twin framework	Continuous model updating and predictive simulation	[41, 51]
World models	Generalized world models for model‐based control	Model‐based reinforcement learning	Pretraining generalized dynamics and fine‐tuning for specific deployments	Autonomous navigation	[23, 66, 67]
Clinical translation	AI‐assisted multimodal imaging for microrobotics	Deep learning and multimodal imaging	Precision medicine	Real‐time localization and intervention planning	[32, 65]
Environmental translation	AI‐assisted pollutant and microplastic removal	Machine learning	Environmental cleanup	Multi‐objective optimization of remediation efficiency	[24]

## 6 Translational Applications

### 6.1 Biomedical Applications

One of the most impactful fields of implementation of AI technology with micro‐ and nanorobots is biomedical and translational medicine. Current drug delivery systems in conventional medicine rely on passive targeting mechanisms such as enhanced permeability and retention (EPR) effect, which are limited by low bioavailability. In contrast, micro‐ and nanorobots offer active, adaptive targeting, navigation, sensing, and actuation in real time using different stimuli or propulsion mechanisms [70, 71, 72, 73]. Engaging AI technology with micro‐ and nanorobots introduces a new class of adaptive, self‐learning, and decision‐making devices capable of dynamic actuation and sensing while navigating complex physiological environments [18]. One promising application is targeted drug delivery, where AI‐enhanced micro‐ and nanorobots can autonomously actuate toward tumor regions, inflamed tissues, or thrombosis regions based on the collected data. These approaches enhance bioavailability and minimize systemic toxicity via high‐precision therapeutic cargo delivery.

Another transformative application is minimally invasive medical imaging and biosensing. Micro‐ and nanorobots integrated with acoustic or magnetic propulsion mechanisms can both navigate and sense simultaneously [32].

### 6.2 Environmental Applications

AI‐assisted micro‐ and nanorobots are also promising for addressing urgent environmental challenges, including the capture and degradation of organic pollutants, forever chemicals, and microplastics. Conventional remediation methods require high energy consumption and are often inefficient. According to current technology, micro‐ and nanorobots may have limitations on large‐scale fabrication. However, AI‐enhanced micro‐ and nanorobots may offer scalable and adaptive alternatives. The removal of microplastics and organic pollutants from the aquatic environment by machine learning‐incorporated magnetic micro‐ and nanorobots has been reported where removal efficiency reached up to 96%. Machine learning was used for multi‐objective optimization [24].

## 7 Conclusion and Outlook

We foresee that advances in multimodal AI will drive micro‐ and nanorobots toward Level 4–5 autonomy within the next decade (Figure 5), representing a transition from operator‐dependent architectures to fully autonomous systems. Incorporating AI‐technology for autonomous navigation, multimodal imaging, biocompatible materials, and digital twin integration to function in vivo will enable the development of patient‐specific micro‐ and nanorobots. It is initially required to collect biologically relevant data, create digital twin models, follow semi‐autonomous to closed‐loop autonomous micro‐ and nanorobots, and, finally, swarms of self‐learning, adaptive agents. This trajectory aligns with the evolution of autonomous vehicles, but the impact on public health and environmental safety would be revolutionary.

**FIGURE 5:**
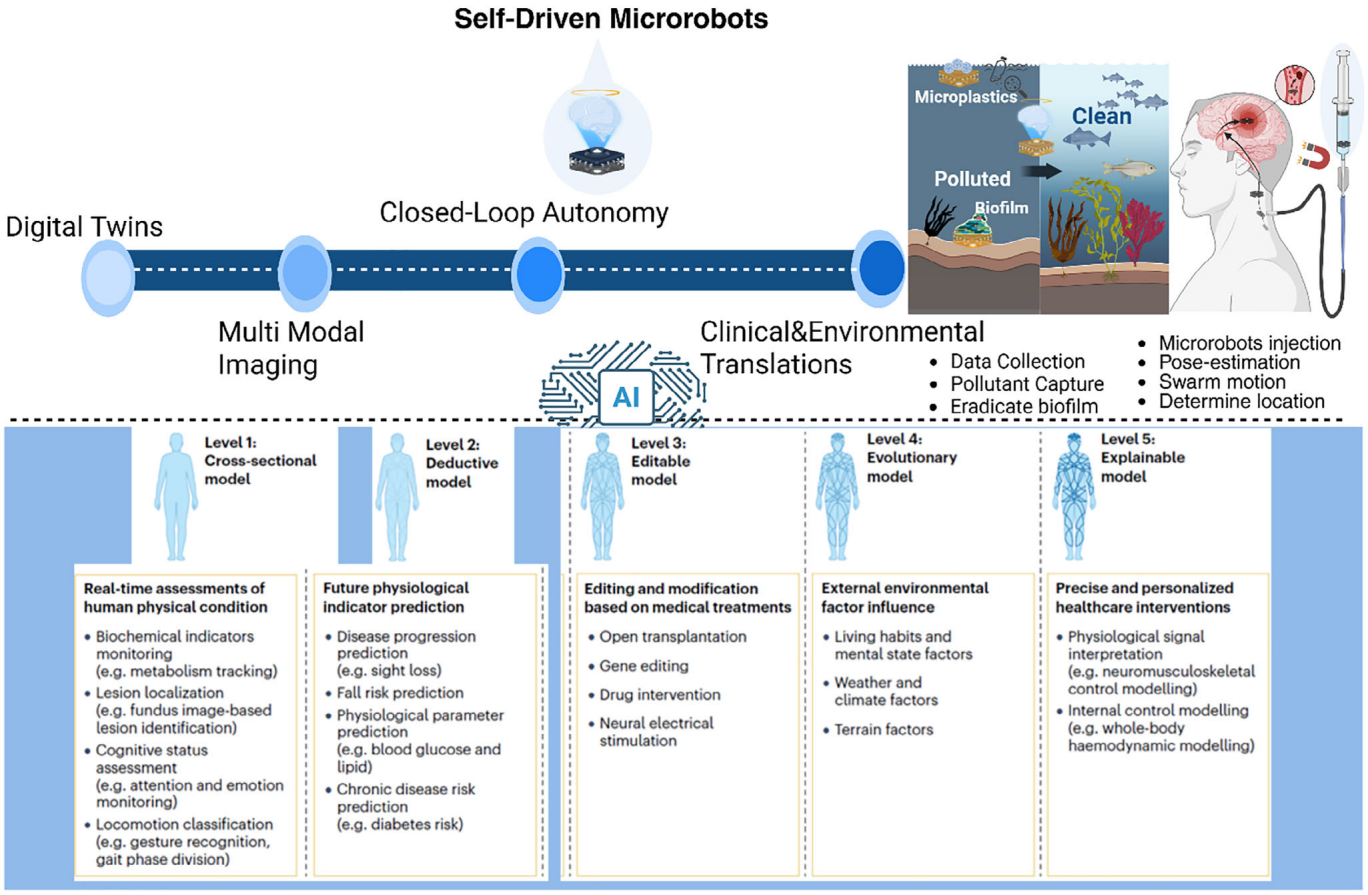
Roadmap of AI‐assisted micro‐ and nanorobot technologies into clinical and environmental translations. Different levels of digital twin models with AI algorithms from Level 1 to Level 5. Reproduced with permission [74]. Copyright 2024, Springer Nature Limited. While digital twins provide close‐to‐precise information via models, multimodal imaging will assist in moving toward closed‐loop autonomy in micro‐ and nanorobotic technology [75]. Inc. https://BioRender.com/8d32dxb.

The convergence of AI and micro‐ and nanorobots is the beginning of a new era for translational medicine and environmental science, where autonomous, intelligent machines operate at the smallest scales with self‐learning and decision‐making skills while micro‐ and nanorobots can interact with the environment and determine routes based on previous experience. Figure 5 presents a roadmap starting with the development of digital twins through to the goal of clinical and environmental translation. Digital twin models provide individual organs and tissues of the human body to determine real‐time physical and biochemical status at Level 1. Level 2 provides detailed information and uses real‐time and past data to train predictive models. Level 3 determines the simulation of interventions and drug administration, or gene editing, before real interventions. Level 4 incorporates information from external factors to make precise decisions and Level 5 performs physiological signal interpretation. Each level of digital twin modeling enhances the precise delivery and self‐decision‐making of micro‐ and nanorobots. The integration of AI‐driven multimodal imaging with micro‐ and nanorobotic systems is expected to enhance how effectively we gather and combine correlative, complementary, and distinguishing information from various imaging techniques for their precise actuation and delivery. Next, the optimization of closed‐loop control of micro‐ and nanorobots will allow precise, fully autonomous, and obstacle‐free actuation. The goal is the application of AI‐assisted, fully autonomous self‐decision‐making micro‐ and nanorobots into clinical and environmental translational studies.

Recent developments in machine learning, reinforcement learning, and data‐driven design have transforming open‐loop micro‐ and nanorobots into closed‐loop, fully autonomous systems, transitioning them from externally guided micro‐ and nanorobots to adaptive, self‐learning systems capable of precision navigation, swarm coordination, and multimodal sensing. It is envisioned that there will be a transition from proof‐of‐concept studies to clinically and environmentally relevant applications. AI provides both biomedical and environmental applications with real‐time adaptive autonomy, continuous learning, and closed‐loop control, reducing dependency on external operators. This capability will enable clinicians to guide micro‐ and nanorobots with minimal manual input while maximizing precision. In environmental applications, it could lead to self‐deploying remediation systems that operate autonomously in remote or dynamic ecosystems, including living organisms via fluidics. However, several challenges remain, including ensuring safety and reliability, scalable fabrication, biocompatibility, ethical concerns, a lack of large‐scale data, and regulatory limitations. The safe implementation of AI‐assisted micro‐ and nanorobots can possibly be achieved within verified physical, biological, and operational bounds, pre‐validation using digital twins, and an automatic deactivation option. Future advancement in the field requires interdisciplinary collaboration, including robotics, AI, materials science, and life sciences, before clinical translation.

## Author Contributions

**Michal Otyepka**: conceptualization, writing – original draft, writing – review and editing, supervision, resources. **Hamed Shahsavan**: conceptualization, writing – original draft, writing – review and editing, supervision, resources. **Fatma M. Yurtsever**: conceptualization, writing – review and editing, writing – original draft. **Minitha Cherukutty Ramakrishnan**: conceptualization, writing – original draft, writing – review and editing. **Ian Kuula Ross**: writing – original draft, writing – review and editing. **Ivan Zelinka**: writing – original draft, writing – review and editing. **Martin Pumera**: conceptualization, writing – original draft, funding acquisition, writing – review and editing, resources, supervision.

## Conflicts of Interest

M.P. joined the Scientific Advisory Board of Selene Nanorobotics Inc, USA, after the completion and initial submission of this work and holds stock options in the company. M.P. cofounded Spintuo s.r.o., Czech Republic, after the completion and initial submission of this work. These relationships arose only after the submission of the manuscript and had no role in the conception, design, execution, analysis, interpretation, or preparation of the article. M.P. discloses these interests in the interest of full transparency. The other authors declare no conflicts of interest.

## Data Availability

The data that support the findings of this study are available from the corresponding author upon reasonable request.
